# Morgagni's diaphragmatic hernia mimicking a severe congenital heart disease in a newborn: a case report

**DOI:** 10.1186/1752-1947-4-395

**Published:** 2010-12-08

**Authors:** Pier Paolo Bassareo, Paola Neroni, Sabrina Montis, Roberto Tumbarello

**Affiliations:** 1 Division of Paediatric Cardiology, Giuseppe Brotzu Hospital, P.le Alessandro Ricchi 1, 09100 Cagliari, Italy

## Abstract

**Introduction:**

Morgagni's congenital diaphragmatic defect is a rare malformation, the diagnosis of which, as in our case report, may be problematic. To the best of our knowledge, this is the first report of this kind of hernia presenting with signs and symptoms of severe cardiac malformation.

**Case presentation:**

We report the case of a three-month-old Caucasian baby boy, who presented with heart failure and severe pulmonary hypertension. Compression of the heart by a bowel loop in the chest led to an incorrect diagnosis of congenital heart disease.

**Conclusions:**

Even in this era of highly sophisticated diagnostic tools, a simple radiograph can provide sufficient information for a precise, rapid diagnosis.

## Introduction

Congenital diaphragmatic hernia (CDH) is a congenital birth defect involving abnormal development of the diaphragm. The condition is produced by a hole in the diaphragm, which allows the abdominal contents to protrude into the chest cavity. Accounting for approximately 2% of all CDH cases, Morgagni's CDH is characterized by herniation through the foramina of Morgagni, located immediately adjacent to the xiphoid process of the sternum [1,2]. The defect in the diaphragm is generally located on the right side (90%) or bilaterally (7%); occasionally it may be on the left side, although the presence of both heart and pericardium are a barrier against herniation [3,4]. Most patients are asymptomatic until adulthood. The lesion rarely presents during the neonatal period, in which case it leads to severe respiratory distress, sometimes associated with anomalies in other organs, including the heart [5,6]. Diagnosis may prove problematic, as we describe in our case report below.

## Case presentation

A three-month-old white Caucasian baby boy, was taken to our emergency department because of breathing difficulties. He was born at 34 weeks of gestation after an uncomplicated vaginal delivery and weighed 2245 g at birth. He had been diagnosed at another hospital as having severe congenital heart disease [hypoplasia of the right ventricle, hypoplasia of the tricuspid valve, and an ostium secundum atrial septal defect (osASD)], widespread calcification of the left lobe of the liver, and 'horseshoe' kidney, for which he was treated with furosemide 1 mg/kg twice daily at the time of presentation. Based on the assumed absence of a normal pumping right chamber, the possibility of surgical intervention (bidirectional Glenn shunt at the age of four to twelve months and possible completion of the Fontan circuit at one to five years of age) was under consideration.

On clinical examination, our patient was cyanotic and tachypnoeic (approximately 80 breaths/min), with oxygen saturation of 78%. He was lethargic and hypotonic. No morphologic alterations to the central nervous system were found.

**Figure 1:**
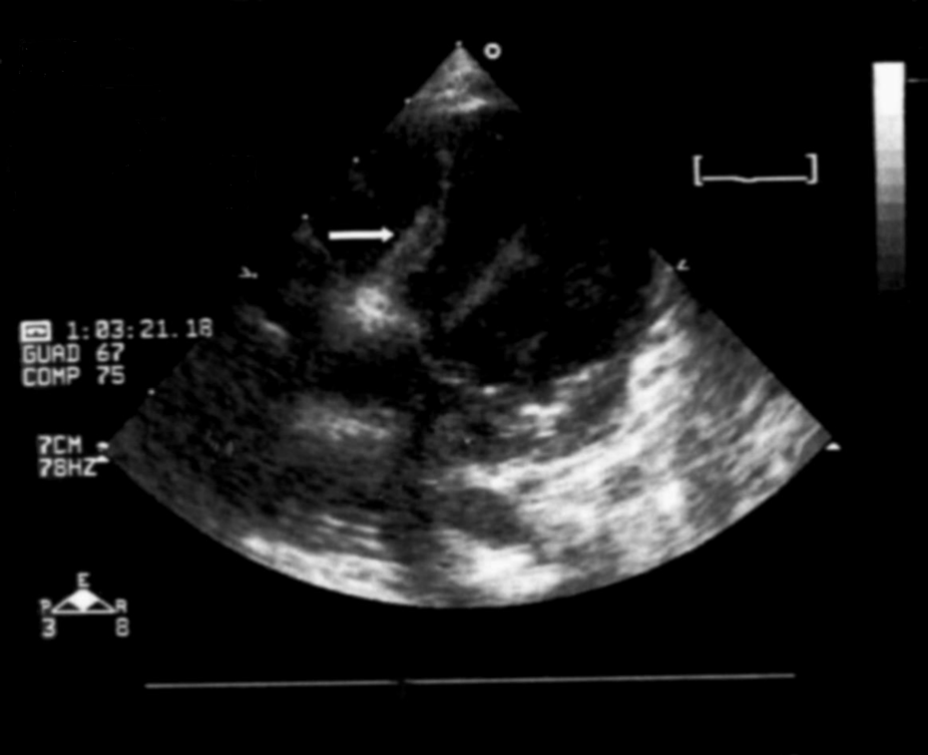
**Echocardiographic apical four-chamber view**. Bowel loops severely compressing the right side of the heart (white arrow).

Electrocardiography revealed sinus tachycardia (120 beats/min), and complete right bundle branch block. Transthoracic echocardiography (Figure 1) revealed a mild pericardial effusion, with the heart shifted to the left, and a wide (12 mm) osASD with bidirectional shunting; hypoplasia of both the right ventricle and tricuspid valve was also confirmed. Additionally, a moderate right outflow obstruction was detected. Pulmonary pressure was calculated by Doppler sonography from the tricuspid insufficiency, and had almost systemic values (65 mmHg). Moreover, the hypocontractile anterior wall of the right ventricle was compressed by an anechogenic formation located between heart and sternum. Furthermore, owing to the absence of a clear connection between the right pulmonary vein and the left atrium, an anomalous partial pulmonary venous drain was suspected.

**Figure 2:**
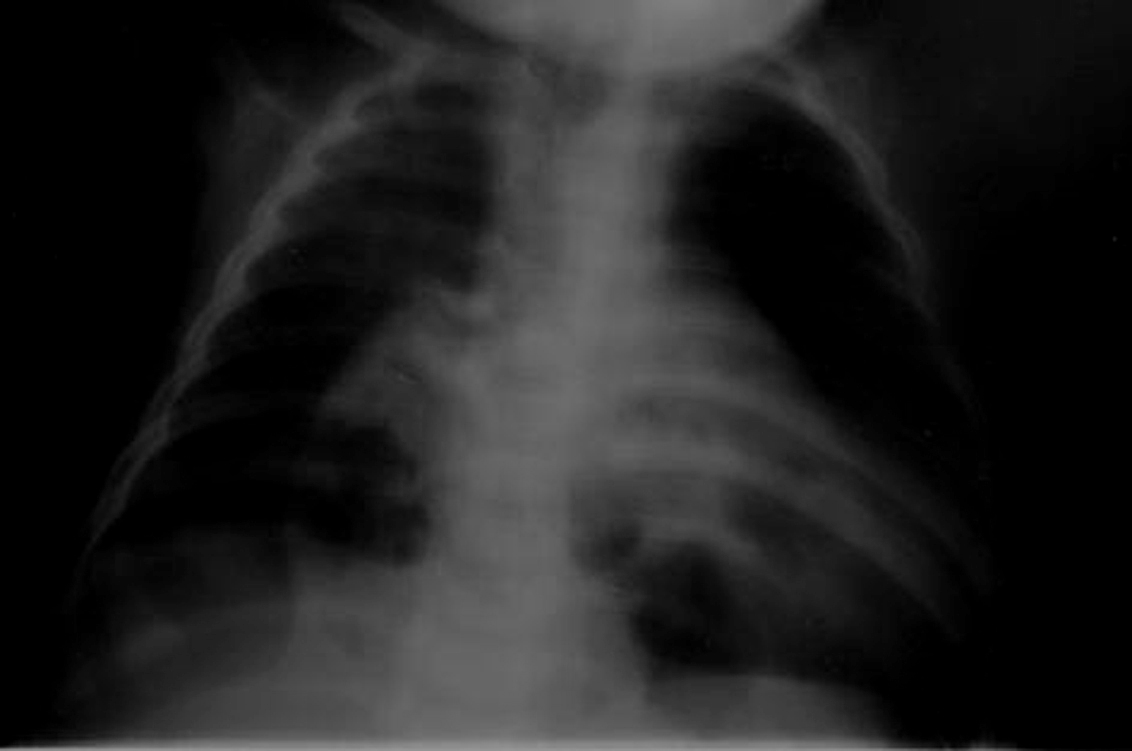
**Posteroanterior chest X-ray**. Bowel loops compressing the right ventricle of the heart.

**Figure 3:**
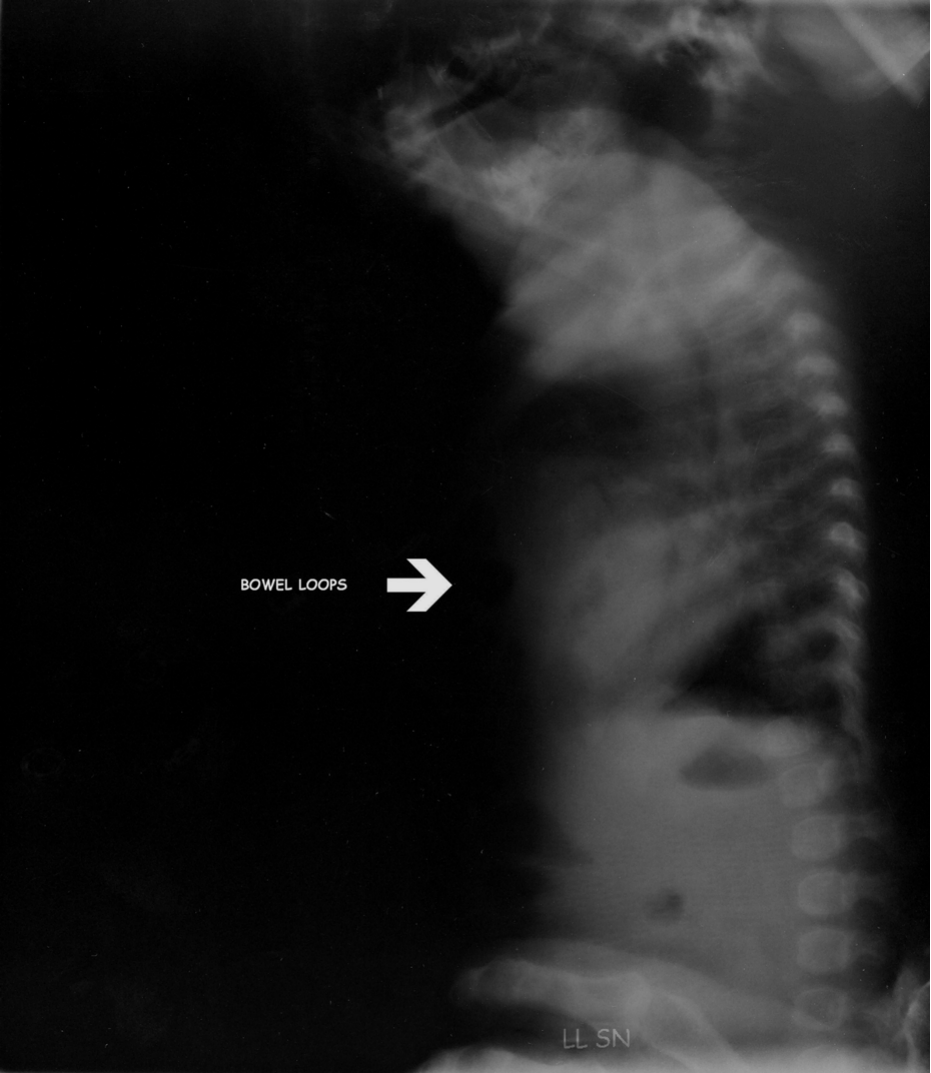
**Lateral chest X-ray**. Bowel loops compressing the right ventricle of the heart (white arrow).

**Figure 4:**
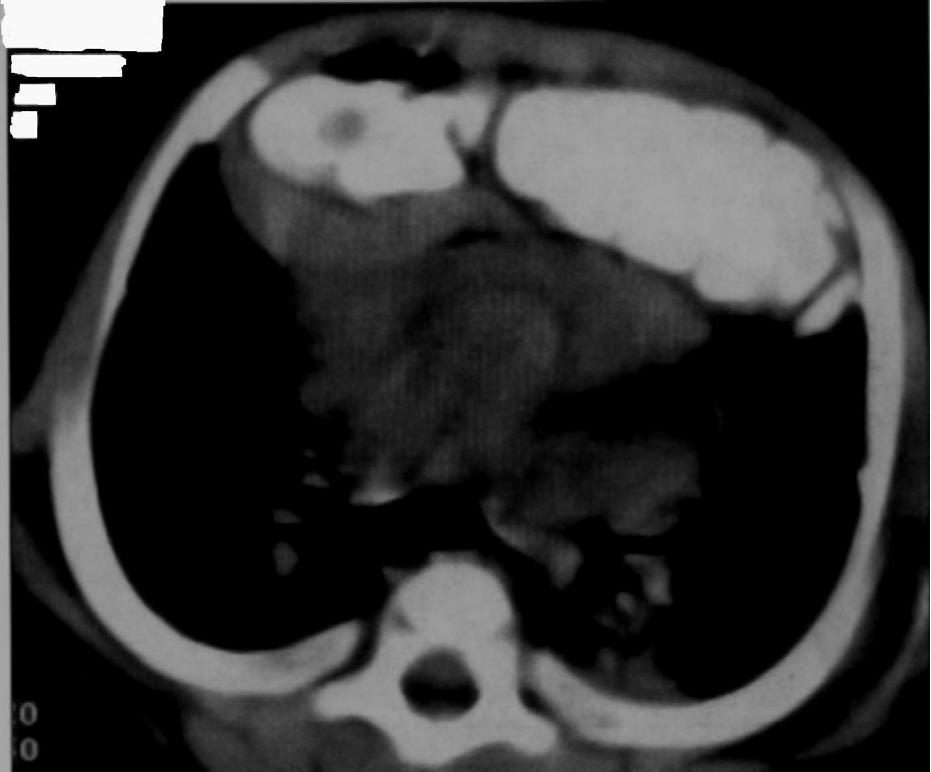
**Computed tomography scan of the chest**. The abdominal contents pass into the anterior mediastinum and compress the right side of the heart,

A posteroanterior and lateral chest X-ray showed the presence of bowel loops in the chest with mediastinal shift and compression on the right ventricle (Figure 2 Figure 3). Computed tomography of the chest confirmed this diagnosis, showing the abdominal contents passing through an anterior diaphragmatic defect into the anterior mediastinum and severely compressing the right side of the heart (Figure 4).

**Figure 5:**
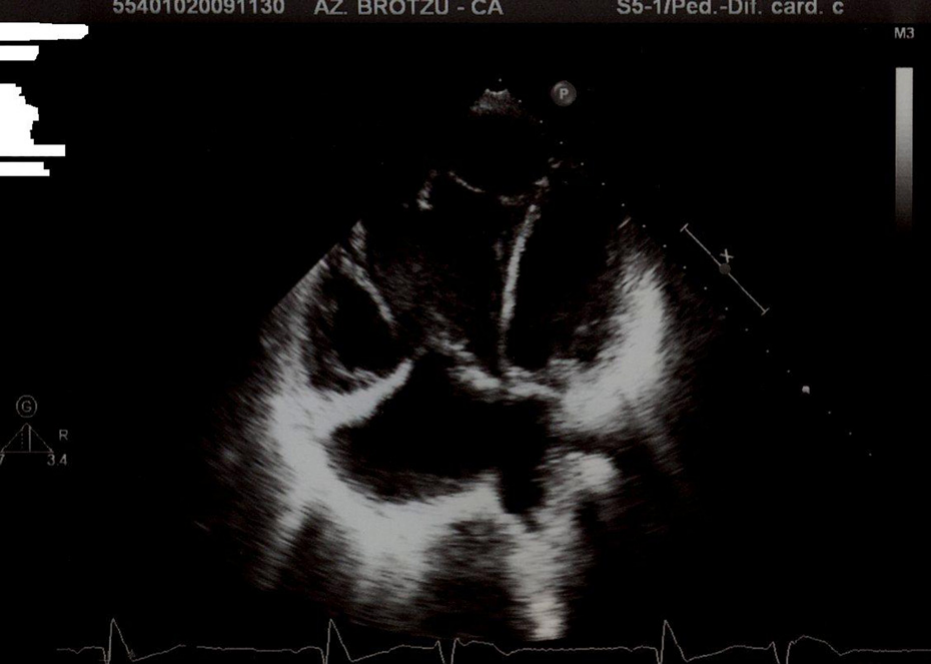
**Echocardiographic apical four-chamber view after surgery**. Normalization of both the right ventricle and tricuspid valve size. A wide atrial septal defect type ostium secundum is also evident.

The child was successfully treated by laparoscopic surgery. Follow-up echocardiography was consistent with normalization of pulmonary pressure, a normal right ventricle and tricuspid valve, and a persistent osASD with unidirectional left to right shunt (Figure 5). The child was discharged two weeks after surgery in excellent clinical condition and with an oxygen saturation of 98%.

## Discussion

Morgagni's congenital diaphragmatic defect, a rare anterior defect of the diaphragm described for the first time by Morgagni in 1761, is frequently asymptomatic and is generally diagnosed incidentally during the investigation of other conditions [7]. Where symptoms are present, these are usually due to compression of thoracic organs. Accordingly, patients may present with respiratory distress. Additionally, recurrent chest infections and gastrointestinal symptoms (vomiting, abdominal pain and bleeding) have been reported in subjects with previously undiagnosed Morgagni's hernia. The rarity of this CDH and the non-specific symptoms may lead to a delay in diagnosis, particularly in childhood [5,8].

Our patient was in seemingly good clinical health at birth and during his first three months of life. His chest X-ray at birth was reported as normal, probably because the abdominal bowel loops were not yet herniated through the foramina of Morgagni in the diaphragm. At about three months, the patient developed acute respiratory distress. He was examined first in a small suburban hospital, where the incorrect diagnosis of congenital heart disease was posed based on his echocardiography findings. It was reported in the patient's notes that chest radiography had been performed, but possibly because of the urgency in transferring him to our centre, the radiological diagnosis was not reported.

Only two cases of a Morgagni's CDH involving the pericardium and mimicking an intrapericardial tumour have previously been reported in the literature. Both cases were diagnosed by fetal echocardiography [9]. In this case report, our patient, born with Morgagni's CDH, showed severe respiratory distress due to both pulmonary hypertension and pulmonary hypoplasia. The first condition is a restriction of blood flow through the lungs, thought to be caused by both external compression and anomalies in the pulmonary alveoli, frequently encountered in this condition. Pulmonary hypoplasia, or decreased lung volume, is directly related to the presence of abdominal organs in the chest cavity, which makes the lungs severely undersized, particularly on the side of the hernia. In addition, compression of the right ventricle from the front mimicked echocardiographic findings, suggesting the presence of right ventricular hypoplasia, a rare life-threatening congenital heart disease. The so called 'hypoplastic right heart syndrome' refers to an underdevelopment of the structures on the right side of the heart, which causes inadequate blood flow to the lungs, and consequently cyanosis. Features include a very small or hypoplastic right ventricle (lower chamber which normally pumps blood to the lungs) and a small tricuspid valve. Atresia of the pulmonary valve and hypoplastic pulmonary artery are usually associated with this condition [10]. The diagnosis of anomalous partial venous drainage was based on both the compression of the atria and consequent dislocation of the right pulmonary veins.

## Conclusions

Morgagni's CDH is a rare malformation, which is difficult to diagnose by even the most experienced physicians. In this previously unreported case, compression of the heart by the bowel might have led to the incorrect diagnosis of congenital heart disease. Even in this era of highly sophisticated diagnostic tools, a simple radiograph can provide sufficient information for a precise, rapid diagnosis.

## Competing interests

The authors declare that they have no competing interests.

## Authors' contributions

PPB carried out the acquisition of data, conception and design. PN was a major contributor to writing the manuscript. SM critically revised the manuscript critically. RT had final approval of the version to be published. All authors have read and approved the final manuscript.

## Consent

Written informed consent was obtained from the patient's parents for the publication of his case report and any accompanying images. A copy of the written consent is available for review by the Editor-in-Chief of this journal.
